# Purification of Low‐Complexity Domain Proteins FUS, EWSR1, and Their Fusions

**DOI:** 10.1002/cpz1.70136

**Published:** 2025-04-26

**Authors:** Jesse J. Altemus, Michelle A. Lay, Valery F. Thompson, Jacob C. Schwartz

**Affiliations:** ^1^ Department of Pharmacology University of Arizona College of Medicine Tucson Arizona; ^2^ University of Arizona Cancer Center Tucson Arizona; ^3^ Department of Chemistry and Biochemistry University of Arizona Tucson Arizona; ^4^ These authors contributed equally to this work

**Keywords:** Ewing sarcoma, fusion protein, low‐complexity domain, neurodegenerative disease, phase separation, protein purification

## Abstract

FET proteins are large multifunctional proteins that have several key roles in biology. The FET family of proteins, including FUS, EWSR1, and TAF15, play critical roles in transcription regulation, RNA processing, and DNA damage repair. These multifunctional RNA‐ and DNA‐binding proteins are ubiquitously expressed and conserved across vertebrate species. They contain low‐complexity (LC) domains that allow them to assemble and phase separate but also makes the proteins prone to aggregation. Aberrations in FET proteins, such as point mutations, aggregation, or translocations leading to fusion proteins, have been implicated in several pathologies, including frontotemporal lobar degeneration (FTLD), amyotrophic lateral sclerosis (ALS), and Ewing sarcoma. In vitro study of FET proteins is hampered by their propensity to aggregate, their disordered structure, and their susceptibility to proteolysis, making high‐yield production difficult. Here, we present optimized methods for the purification of full‐length FUS, EWSR1, and their fusion proteins. These protocols enable researchers to overcome issues related to aggregation and solubility, facilitating biochemical and biophysical studies of these critical yet complex proteins. © 2025 The Author(s). Current Protocols published by Wiley Periodicals LLC.

**Basic Protocol**: Purification of EWSR1 and FUS proteins

**Alternate Protocol**: Purification for fusion proteins

## INTRODUCTION

Precise regulation of transcription, gene expression, and RNA processing is critical to maintaining healthy cellular function (Cech, 2022). Many proteins contribute to these processes, among which are the FET (FUS, EWSR1, and TAF15) family of proteins (Schwartz et al., 2015). This prominent family of multifunctional RNA binding proteins are ubiquitously expressed across metazoan organisms (Kawaguchi et al., 2020; Ozdilek et al., 2017). Point mutation, aggregation, or fusion of FET proteins have been shown to cause diseases, such as frontotemporal lobar degeneration (FTLD), amyotrophic lateral sclerosis (ALS), and Ewing sarcoma (Purice & Taylor, 2018; Riggi et al., 2021; Tetter et al., 2024). The third leading genetic cause for ALS is point mutations in the FUS protein, with >50 FUS mutations identified so far (Al‐Chalabi et al., 2024; Goldstein et al., 2022). These mutations lead FUS aggregates to form in motor neurons and produce cell toxicity. FUS aggregates are also found in FTLD, essential tremor, and basophilic inclusion body disease (BIBD) (Purice & Taylor, 2018). In pathologies where FUS aggregates are found, other FET and low‐complexity (LC) domain proteins may be co‐aggregates (Kawaguchi et al., 2020; Reber et al., 2021). FET proteins have been challenging to isolate for in vitro assays, primarily because the LC domain tends to drive aggregation and leads to irreversible insolubility (Kumar et al., 2023; Murray et al., 2017; Selig et al., 2023). Furthermore, disordered domains in these proteins are often targeted by proteases due to exposed residues along flexible loops (Schuster et al., 2018). These properties have limited the ability to purify substantial amounts of these proteins, thereby necessitating a novel approach for their purification to facilitate the study of this critical family of proteins (K. M. Johnson et al., 2017; Selig et al., 2023).

**Figure 1:**
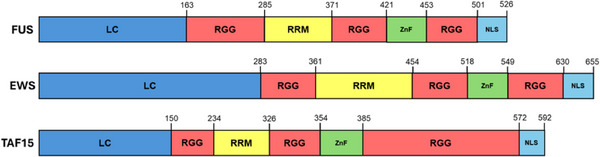
Domains of FET proteins. Diagram of the domains in FET proteins. LC domain, low‐complexity domain; RGG, arginine/glycine/glycine‐rich domain; RRM, RNA‐recognition motif; ZnF, zinc‐finger domain; NLS, nuclear localization signal.

FET proteins are mostly comprised of disordered domains containing low‐complexity amino acid sequences (Fig. 1) (C. N. Johnson et al., 2024; Thompson et al., 2018). Repeated motifs of tyrosine, serine, glycine, and glutamine residues make up the N‐terminal LC domain (Kato et al., 2022; Murray et al., 2017) that facilitates protein–protein interactions and self‐assembly (Zhou et al., 2022). Arginine and glycine residue repeats are enriched in the RGG domains that bind nucleic acids and contribute to FET protein assembly properties (Lay et al., 2024; Ozdilek et al., 2017). In addition to these disordered domains, FET proteins possess an RNA‐recognition motif (RRM) with a unique β‐hairpin structure and non‐canonical RNP1 sequence that is highly conserved across the FET family (Loughlin et al., 2019). The final domain of the FET protein is a zinc finger domain (ZnF) with the zinc coordinated between four cysteines that facilitates binding to single‐stranded nucleic acids (Loughlin et al., 2019; Schwartz et al., 2015; Selig et al., 2023).

FET proteins are capable of binding both RNA and DNA, which distinguishes them from many other nucleic acid‐binding proteins (Lay et al., 2024; Schwartz et al., 2015; Selig et al., 2023). This dual binding ability can be vital for their roles in essential processes like DNA damage repair and RNA processing. Studies have shown FUS and EWSR1 can bind to secondary structures, such as RNA stem‐loops and G‐quadruplex (G4) DNA, further underscoring the functional diversity and specificity of FET proteins in nucleic acid interactions (Lay et al., 2024; Loughlin et al., 2019; Ozdilek et al., 2017; Selig et al., 2023). Recent studies highlight interactions with R‐loops, composed of hybridized DNA–RNA plus a displaced DNA strand (ssDNA) (Hill et al., 2016; Pan et al., 2020). FUS is instrumental in preventing R‐loop formation by binding to nascent RNA (Thompson et al., 2023). EWSR1 specifically recognizes DNA fork structures at the boundaries of R‐loops (Lay et al., 2024; Pan et al., 2020). TAF15 has been observed in nucleoli condensates upon inhibition of RNA Polymerase II and interacts with EWSR1, unphosphorylated RNA polymerase II, spliceosome components, and auxiliary proteins (Chi et al., 2018; Thomsen et al., 2013; Yasuhara et al., 2022).

With such a wide array of critical functions, aberrations in FET proteins can lead to many pathologies. FUS and TAF15 can form amyloid filaments observed in FTLD, though mutations in these genes have not been reported for this disease (Tetter et al., 2024). Translocations of FUS or EWSR1 can generate a large number of fusion proteins that drive several sarcomas, with the most notable being Ewing sarcoma. Sarcoma‐driving fusions mostly involve an E28 transformation‐specific (ETS) family gene (Nacev et al., 2020; Riggi et al., 2021). EWS‐FLI1 is the most common fusion protein formed by a translocation of *EWSR1* and *FLI1* genes and is responsible for >85% of Ewing sarcoma tumors (Table 1). Other Ewing sarcoma cases are caused by alternative ETS protein fusions, including EWS‐ERG, EWS‐ETV1, EWS‐ETV4, EWS‐ETV5, EWS‐FEV, FUS‐FEV, and FUS‐ERG (Boone et al., 2021; Grunewald et al., 2018). Tumors in patients with these fusions are morphologically and pathologically like those with the EWS‐FLI1 fusion (Grunewald et al., 2018). The FUS‐ERG fusion occurs primarily in acute myeloid leukemia patients, though rarely it has been seen in myeloid sarcoma tumors and Ewing sarcomas (Buchanan & Tirado, 2016; Chen et al., 2021; Ueda et al., 2016). Gene rearrangements of EWSR1 or FUS have also been shown to drive other sarcomas, including neuroectodermal tumor (ZNF278 and POU5F1), myxoid liposarcoma (DDIT3), clear cell sarcoma (ATF1 and CREB1), desmoplastic small round cell sarcoma (WT1), extra skeletal myxoid chondrosarcoma (NR4A3), hidradenoma (POU5F1 and PBX1), mucoepidermoid carcinoma (POU5F1), and leukemias (ZNF384) (Grunewald et al., 2018; Riggi et al., 2007, 2021).

**Table 1:** FET‐ETS Gene Fusions and Their Associated Tumors

Tumor type	FET proteins	Fusion partner
Ewing sarcoma	FUS, EWSR1	Fli1, ERG, ETV1, ETV4, ETV5
NFATc2‐rearranged sarcoma	FUS, EWSR1	NFATc2
Desmoplastic small round–cell tumors	EWSR1	WT1
Myxoid liposarcoma	FUS, EWSR1	DDIT3
Angiomatoid fibrous histiocytoma	FUS, EWSR1	CREB, ATF
Intracranial mesenchymal tumors	EWSR1	ATF1, CREB1, CREM
Primary pulmonary myoxid sarcoma	EWSR1	CREB1
Acute myeloid leukemia	FUS, TAF15, EWSR1	FEV, ERG, ZNF384
Mesothelioma	FUS, EWSR1	CREB
Myoepithelioma	EWSR1	PBX1, PBX3, ZNF444, POU5F1, ATF1, KLF17
Sclerosing epithelioid fibrosarcoma	FUS, EWSR1	CREB3L1, CREB3L2, CREB3L3, CREM
Extraskeletal myxoid chondrosarcoma	EWSR1, TAF15	NR4A3
EWSR1‐SMAD3‐positive fibroblastic tumor	EWSR1	SMAD3
Epithelioid and spindle cell rhabdomyosarcoma	FUS	TFCP2

The lack of intramolecular interactions in a disordered protein can facilitate intermolecular interactions with other proteins and nucleic acids (Cai et al., 2021; Forman‐Kay et al., 2022; Kato et al., 2022). A biological function of FET proteins involves assembly through its disordered domains, which leads to phase separation (C. N. Johnson et al., 2024; Schwartz et al., 2015). Phase separation by FET proteins results in a condensate with liquid‐like properties, a process termed liquid–liquid phase separation (LLPS). Outside of its ordinary functioning environment, the proteins are prone to phase separate by LLPS or form solid hydrogels and aggregates (Forman‐Kay et al., 2022; Kato et al., 2022). This is a significant consideration while working with the recombinant protein since disordered FET proteins expressed recombinantly tend to enter inclusion bodies in *Escherichia coli* and may become insoluble during purification.

## STRATEGIC PLANNING

### Urea to prevent RNA binding and aggregation

Proteins with intrinsically disordered domains are prone to aggregation during purification (Cech, 2022; Shevtsov & Dundr, 2011). Increasing concentration of a mild denaturing agent, such as urea, helps the protein remain soluble (Lay et al., 2024; Schwartz et al., 2013; Victor et al., 2021). The method described here was developed to accommodate the aggregation and phase separation properties of FET proteins. While a propensity to aggregate is unfortunate for purposes of purification and storage of recombinant protein, it is this activity that underlies normal FET protein activity and their related diseases. We utilize urea (1 M) as a denaturing reagent to prevent intermolecular interaction and to aid FET proteins solubility. It should be noted that studies that denature proteins using urea usually use 8 M concentrations and 1 M urea generally does not cause significant unfolding at physiologic or room temperatures (Canchi et al., 2010; Nikolova et al., 1998). Concentrations of urea below 4 M have not been found to denature RRM or ZnF domains in other proteins (Zaharias et al., 2021; Zhao & Huang, 2016). Additionally, we added a 6×His‐MBP tag to the N‐terminal of the protein to improve protein solubility and for affinity purification. In a previous study, we found that 4 M urea had only a small effect on MBP structure that facilitates bioconjugation of residues near the surface (Moinpour et al., 2020). Our early attempts at purification of denatured FUS proteins were unsuccessful; a more recent study suggests that this was due to the propensity of the RRM domain to collapse into amyloid aggregates rather than to refold (Lu et al., 2017).

Co‐purification of RNA‐binding proteins with RNA molecules and other *E. coli* proteins almost always occurs. RNA is a sticky molecule that can bind a wide variety of proteins; this further increases the probability of aggregation during purification. The steps in these protocols that minimize co‐purification of nucleic acids include addition of the nuclease benzonase and polyethyleneimine (PEI) to sediment nucleic acids and cell debris. Alternatively, for studies in which residual nuclease activity is of significant concern, micrococcal nuclease (MNase) can be added after affinity purification or size‐exclusion chromatography (Schwartz et al., 2012, 2013). MNase is easily inactivated by EGTA.

### Protein tags for solubility

Insolubility of FET proteins is well known and use of a tag to improve solubility and to slow phase separation is common (Table 2). The maltose‐binding protein (MBP) is an excellent choice of tag for this purpose. MBP is a 40.3 kDa well‐structured and globular protein. Another option is glutathione S‐transferase (GST). A GST tag has been used by previous studies to maintain FET proteins in their monomer state until cleavage, which triggers rapid assembly and phase separation. We have successfully purified FUS and EWSR1 with a GST fusion tag. In our experience, GFP and mCherry tags promote solubility for the LC domain of FUS and modestly promote solubility to full length FUS. Other fusion tags that our lab has tested for FUS protein that offered modest or no improvement to solubility include SUMO, SNAP, 6×His, and FLAG.

**Table 2:** Protein Properties for FUS, EWSR1, and Fusions*^a^*

Protein	Uniprot #/a.a. boundary	# amino acids	Predicted MW (kDa)	Observed MW (kDa)	Theoretical pI
FUS	***P35637***	526	53.3	75	9.4
H‐MBP‐FUS	a.a. 1‐526	920	96.7	110‐120	7.7
H‐MBP‐FUS‐LC	a.a. 1‐167	557	60.4	60	5.1
EWSR1	***Q01844***	656	68.5	90	9.4
H‐MBP‐EWSR1	a.a. 1‐656	1050	111.7	140‐150	8.2
H‐MBP‐EWSR1‐delLC	a.a. 290‐656	760	81.3	80	8.9
H‐MBP‐EWS‐R_1_‐RRM‐R_2_	a.a. 290‐515	621	66.6	70	6.0
H‐MBP‐EWS‐R_2_‐ZnF‐R_3_	a.a. 454‐656	597	63.9	75	9.1
EWS‐FLI1	***B1PRL2***	498	54.3	70	8.5
H‐MBP‐EWS‐FLI1	EWS: a.a. 1‐264 FLI1: a.a. 218‐452	892	97.6	110	6.0
H‐MBP‐EWS‐ETV1	EWS: a.a. 1‐264 ETV1: a.a. 265‐427	822	90.5	N.D.	5.2
H‐MBP‐EWS‐ETV1 (trunc)	EWS: a.a. 1‐264 ETV1: a.a. 265‐383	778	85.4	100	5.4
H‐MBP‐EWS‐ETV1_LCdead	EWS: a.a. 1‐264 ETV1: a.a. 265‐427	822	87.7	120‐130	5.2
H‐MBP‐EWS‐ETV1 DBDnull	EWS: a.a. 1‐264 ETV1: a.a. 265‐427	822	90.3	110‐120	5.1

^*a*^ MW, molecular weight; a.a., amino acid; N.D., not determined.

Both MBP and GST can be used for affinity purification using amylose or glutathione beads, respectively, but they are less efficient at binding and elution and are more expensive. In both respects, a superior option is immobilized metal affinity chromatography (IMAC) using a 6×His tag and Ni‐NTA (nickel‐nitrilotriacetic acid) beads. Our studies have relied on combinations of 6×His‐MBP or 6×His‐GST. Of significant consideration in the use of affinity purification for FET proteins is the high concentrations created by binding to beads that promotes their aggregation. To mitigate this problem, the volume of beads used should be well below the maximum binding capacity for the protein. An optimal ratio can be determined empirically.

There is no simple basis to choose whether protein tags should be fused to the N‐terminal or C‐terminal of a FET or FET fusion protein. Most published studies have used N‐terminal fused tags (Kumar et al., 2023; Lay et al., 2024; Yoshizawa et al., 2018). However, C‐terminal tags have been used in studies of both FET and FET‐fusion proteins, as well as tags fused at both termini (Rhine et al., 2020). It is worthwhile to consider including a protease linker between the protein and the tag. The tobacco etch virus (TEV) protease linker sequence (ENLYFQ↓G) is one popular choice. All our constructs have included a PreScission protease linker (LEVLFQ↓GP) to facilitate the cleavage of FUS or EWSR1 from the 6×His‐MBP tag.

## PURIFICATION OF EWSR1 AND FUS PROTEINS

This protocol was developed and optimized for FUS protein and since has been found to be effective for EWSR1 and truncations and mutants of both these proteins (Table 2). For many cases, Basic Protocol is as effective for FET fusion proteins as any other protocol. However, modifications to this approach described in Alternate Protocol may improve yields. The primary variables here, such as concentration of urea and KCl, are what we have found to be optimal for yield. We may reduce those by half in the elution buffer #1 for protein that is intended to be used in electrophoretic mobility shift assays (EMSA).

### Materials

Chemically competent *E. coli* BL21 cells (NEB, cat. no. C2530H)
Ice
100 ng plasmid DNA appropriate for the protein expression intended
LB growth medium (Miller's) (RPI, cat. no. L24022)
LB agar plates with appropriate antibiotics (RPI, cat. no. L24022; VWR, cat. no. 25384‐088)
Appropriate antibiotic stocks for the DNA plasmid selected
Isopropyl β‐D‐1thiogalactopyranoside (IPTG) (Goldbio, cat. no. I2481C5)
Liquid nitrogen
Lysis buffer #1 (see recipe)
Ni‐NTA beads (GE Healthcare, cat. no. 17‐5268‐02)
Wash buffer #1 (see recipe)
Elution buffer #1 (see recipe)

Programmable heating block
Shaking incubator
15‐ml culture tubes
Incubator, 37°C
250‐ml baffled flasks (Corning, cat. no. 4450‐250)
4‐L baffled flask
Cuvettes
Spectrophotometer
Weighing scale
Floor centrifuge, 4°C
Q125 sonicator with standard probe (Genesee Scientific), or equivalent
Benchtop microcentrifuge (Eppendorf 5424, 5424 R, or equivalent), 4°C
Benchtop centrifuge (Beckman Coulter, Allegra X30R, or equivalent), 4°C
50‐ml conical tubes
Tube rotator
Econo‐Pac chromatography column (Bio‐Rad, cat. no. 7321010)
Epoch2 microplate reader (Thermo Fisher), or equivalent

### Transformation of chemically competent E. coli cells

1 Preheat heating block to 42°C and shaking incubator to 37°C.
2 Thaw 50 µl chemically competent *E. coli* cells for 30 min on ice.
3 Add 100 ng plasmid DNA to cells, flick to mix, and incubate for 30 min on ice.
4 Heat shock cells at 42°C for 45 s.
5 Incubate cells on ice for 2 min.
6 Transfer 50 µl cells to 15‐ml sterile culture tube containing 950 µl LB medium.
7 Incubate at 37°C for 1 hr with shaking at 200 rpm.
8 Spread 50, 100, and 200 µl cells on 3 separate LB agar plates (with appropriate antibiotics).
9 Incubate plate at 37°C for 16 hr.
10 Store plates at 4°C for up to 1 month.

### Growth of transformed E. coli and protein expression

11 Inoculate a single isolated colony in 50 ml LB medium with appropriate antibiotics in a 250 ml‐baffled flask.
12 Grow at 37°C for 16 hr with shaking at 200 rpm.
13 Transfer 10 ml of overnight growth to 1 L of LB with antibiotics in a 4 L‐baffled flask.
14 Grow at 37°C with shaking at 200 rpm until OD_600_ reaches 0.6 to 0.8.
  Measure OD at 600 nm using cuvettes and a spectrophotometer.
  This step typically takes 2 to 3 hr.
15 Induce expression of protein by adding IPTG for a final concentration of 1 mM.
  Using a scale, we weigh IPTG direct from powder then add to LB medium. Alternatively, 1 M stock can be made, stored at −20°C, then 1 ml is added per 1 L of LB medium.
16 Grow at 17°C for 16 hr.
17 Using a floor centrifuge, harvest cell pellets by centrifuging 20 min at 4000 × *g*, 4°C.
18 Flash freeze cell pellets in liquid nitrogen.
19 Store up to 1 month at −80°C until needed.

### Purification of protein

20 Resuspend *E. coli* pellet in lysis buffer #1.
  We typically do 1:5 weight to volume ratio, e.g., 1 gram of pellet is resuspended in 5 ml lysis buffer.
21 Lyse cells on ice with sonication at 55% amplitude for 5 to 7 cycles. Each cycle is 1 min of sonication—15 s on, 15 s off—with 1 min of rest between cycles.
  Be sure to use optimized sonication conditions for your equipment.
22 Clear lysate by centrifuging with a floor centrifuge for 20 min at 16,000 × *g*, 4°C.
23 Equilibrate 1 ml Ni‐NTA beads with 35 ml wash buffer #1.
24 Clear beads by centrifuging with a floor centrifuge for 2 min at 600 × *g*, 4°C.
25 Repeat for a second wash.
26 In a 50‐ml conical tube, incubate lysate with beads at 4°C with rotation for 1 hr.
27 Using a benchtop centrifuge, collect beads by centrifuging 2 min at 600 × *g*, 4°C.
28 Wash beads 4 times with 50 ml wash buffer #1. Centrifuge with a benchtop centrifuge for 2 min at 600 × *g*, 4°C, to remove the first 3 washes. For the last wash, see step 29.
29 For the final wash, add 20 ml wash buffer #1 and transfer with beads to Econo‐Pac chromatography column. Allow wash buffer to flow through and discard.
30 Incubate beads with 1 ml elution buffer #1 at room temperature for 15 min.
31 Collect elution. Repeat the elution (step 30) at least 2 times or until the apparent eluted protein concentration determined by UV absorption is markedly diminished.

## PURIFICATION FOR FUSION PROTEINS

Basic Protocol can be used for FUS, EWSR1, their truncations, and fusions. This protocol is an alternative to Basic Protocol and is intended for fusion proteins, such as EWS‐FLI1, EWS‐ETV1, and their mutant versions. Some considerations for these types of recombinant proteins are that overexpression and subsequent sequestration in the cell can lead to aggregation due to its disordered LC domain, although the incorporation of the alternate ETS domain, ETV1, reduced this liability. The use of benzonase, along with protease inhibitor, aided with digestion of unwanted nucleic acids, their associated proteins, and proteolysis throughout the protocol. Polyethyleneimine (PEI) addition after sonication contributes to clearance of these cleaved nucleic acid products and cell debris via centrifugation. This protocol utilizes a higher concentration of urea and larger number of Ni‐NTA beads to optimize protein yields for this construct.

### Additional Materials (also see Basic Protocol)

Lysis buffer #2 (see recipe)
Polyethyleneimine (PEI) (Sigma Aldrich, 482595‐100ML)
Wash buffer #2 (see recipe)
Elution buffer #2 (see recipe)

2‐L baffled flask

### Transformation of chemically competent E. coli cells

1 Preheat heating block to 42°C and shaking incubator to 37°C.
2 Thaw 50 µl chemically competent *E. coli* cells for 30 min on ice.
3 Add 100 ng of plasmid DNA to cells, mix by flicking, and incubate for 30 min on ice.
4 Heat shock cells at 42°C for 45 s.
5 Incubate cells on ice for 2 min.
6 Transfer 50 µl of cells to a 15‐ml sterile culture tube containing 950 µl LB medium.
7 Incubate at 37°C for 1 hr with shaking at 200 rpm.
8 Spread 50, 100, and 200 µl cell suspension onto 3 separate LB agar plates (with antibiotics).
9 Incubate plate at 37°C for 16 hr.
10 Store plates at 4°C for up to 1 month.

### Growth of transformed E. coli and protein expression

11 Inoculate a single isolated colony into 50 ml LB medium with antibiotics in a 250‐ml baffled flask.
12 Grow at 37°C for 16 hr with shaking at 200 rpm.
13 Transfer 10 ml of overnight growth to 500 ml LB with antibiotics in a 2‐L baffled flask.
14 Grow at 37°C with shaking at 200 rpm until OD_600_ reaches 0.6 to 0.8.
  This step typically takes 2 to 3 hr.
15 Induce expression of protein with IPTG for a final concentration of 1 mM.
16 Grow at 17°C for 16 hr.
17 Using a floor centrifuge, harvest cell pellets by centrifuging 20 min at 4000 × *g*, 4°C.
18 Flash freeze cells pellets in liquid nitrogen.
19 Store at −80°C until needed, up to 2 months for EWSR1 and 6 months for FUS.

### Purification of protein

20 Resuspend *E. coli* pellet in lysis buffer #2.
  Use a 1:5 weight to volume ratio, e.g., 1 gram of pellet is resuspended in 5 ml lysis buffer.
21 Lyse cells on ice with sonication at 55% amplitude for 5 to 7 cycles. Each cycle is 1 min of sonication—15 s on, 15 s off—with 1 min of rest between cycles.
  Be sure to use optimized sonication conditions for your equipment.
22 Add PEI to a final concentration of 0.15% (v/v) directly to cell lysate to clear nucleic acids and cell debris.
23 Using a floor centrifuge, clear lysate with centrifugation for 20 min at 16,000 × *g*, 4°C.
24 Equilibrate 2 ml Ni‐NTA beads with 35 ml wash buffer #2.
25 Clear beads by centrifuging with a benchtop centrifuge for 2 min at 600 × *g*, 4°C.
26 Repeat for a second wash.
27 Incubate lysate with beads at 4°C with rotation for 1 hr.
28 Collect beads by centrifuging with a benchtop centrifuge for 2 min at 600 × *g*, 4°C.
29 Wash beads 4 times with 50 ml wash buffer #2. Centrifuge with a benchtop centrifuge for 2 min at 600 × *g*, 4°C, to remove the first 3 washes. For the last wash, see step 30.
30 For the final wash, add 20 ml wash buffer #2 and transfer with beads to Econo‐Pac chromatography column. Allow wash buffer to flow through and discard.
31 Incubate beads with 1 ml elution buffer #2 at room temp for 15 min.
32 Collect elution. Repeat 2 to 3 times until concentration reaches 1 mg/ml.

## REAGENTS AND SOLUTIONS

### Elution buffer #1

1 M urea (EMD Millipore, cat. no. 9530‐5KG)
1 M KCl (VWR, cat. no. 97061‐560)
50 mM Tris·HCl, pH 7.4 (Goldbio, cat. no. T‐400‐5)
250 mM imidazole (Genesee, cat. no. 18‐208)
Prepare fresh and maintain at 4°C

### Elution buffer #2

1 M urea (EMD Millipore, cat. no. 9530‐5KG)
1 M KCl (VWR, cat. no. 97061‐560)
50 mM Tris·HCl, pH 7.4 (Goldbio, cat. no. T‐400‐5)
300 mM imidazole (Genesee, cat. no. 18‐208)
Prepare fresh and maintain at 4°C

### Lysis buffer #1

1 M urea (EMD Millipore, cat. no. 9530‐5KG)
1 M KCl (VWR, cat. no. 97061‐560)
50 mM Tris·HCl, pH 8.0 (Goldbio, cat. no. T‐400‐5)
10 mM imidazole (Genesee, cat. no. 18‐208)
5% (v/v) glycerol (Fisher Scientific, cat. no. G334)
1% (v/v) NP‐40 (Research Products International, cat. no. N59000‐500.0)
1.5 mM β‐mercaptoethanol (VWR, cat. no. 97064‐880)
1× protease inhibitor (Thermo Fisher Scientific, cat. no. A32965)
Prepare fresh and maintain at 4°C

### Lysis buffer #2

4 M urea (EMD Millipore, cat. no. 9530‐5KG)
1 M KCl (VWR, cat. no. 97061‐560)
50 mM Tris·HCl, pH 8.0 (Goldbio, cat. no. T‐400‐5)
10 mM imidazole (Genesee, cat. no. 18‐208)
5% (v/v) glycerol (Fisher Scientific, cat. no. G334)
1% (v/v) NP‐40 (Research Products International, cat. no. N59000‐500.0)
1.5 mM β‐mercaptoethanol (VWR, cat. no. 97064‐880)
1× protease inhibitor (Thermo Fisher Scientific, cat. no. A32965)
15 U/ml benzonase (EMD Millipore, cat. no. 70746‐3)
Prepare fresh and maintain at 4°C

### Wash buffer #1

1 M urea (EMD Millipore, cat. no. 9530‐5KG)
1 M KCl (VWR, cat. no. 97061‐560)
50 mM Tris·HCl, pH 8.0 (Goldbio, cat. no. T‐400‐5)
10 mM imidazole (Genesee, cat. no. 18‐208)
5% (v/v) glycerol (Fisher Scientific, cat. no. G334)
Prepare fresh and mainstain at 4°C

### Wash buffer #2

4 M urea (EMD Millipore, cat. no. 9530‐5KG)
1 M KCl (VWR, cat. no. 97061‐560)
50 mM Tris·HCl, pH 8.0 (Goldbio, cat. no. T‐400‐5)
10 mM imidazole (Genesee, cat. no. 18‐208)
5% (v/v) glycerol (Fisher Scientific, cat. no. G334)
15 U/ml benzonase (EMD Millipore, cat. no. 70746‐3)
Prepare fresh and maintain at 4°C

## COMMENTARY

### Expected Outcomes

**Figure 2:**
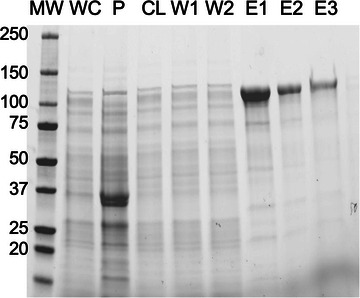
Example of H‐MBP‐FUS protein purification. Lanes from left to right are as follows: molecular weight ladder, MW; whole cell lysate, WC; pellet, P; clarified lysate, CL; washes, W1 and W2; and elutions from Ni‐NTA beads, E1 to E3. Expected weight for H‐MBP‐FUS is 95 kDa and observed is 110 kDa.

For the protein preparation shown in Figure 2, H‐MBP‐FUS was grown overnight as described and pea‐sized pellets of the *E. coli* were flash frozen in liquid nitrogen, then stored at −80°C. The next day 1.8 g of the frozen *E. coli* was thawed and lysed in 25 ml lysis buffer. Affinity purification was performed with 0.5 ml Ni‐NTA beads and 4 washes with 0.5 ml elution buffer yielding between 17 and 60 µM (1.5 to 5.7 mg/ml) in each fraction. Similar preparations of full‐length FUS yield 11 to 100 µM protein in fractions eluted, or 2 to 5.3 mg of total protein per gram of *E. coli* pellet. For H‐MBP‐EWSR1, yields of 0.6 to 2.6 mg protein per gram of *E. coli* pellet were typical, with concentrations in eluted fractions up to 60 µM protein. Truncations of FUS tend to be more soluble and allow higher yields per gram of *E. coli*: 6.5 mg per gram for H‐mCherry‐FUS‐LC, 3.0 mg per gram for H‐GFP‐FUS‐LC, and 1.0 mg per gram for H‐MBP‐del‐LC‐FUS (a.a. 171 to 526). Stock concentrations for del‐LC‐FUS constructs were lower (12 to 58 µM) than for FUS‐LC constructs (100 to 140 µM). Yields and stability of fusion proteins are unavoidably low and poor (Table 3).

**Table 3:** Summary of Protein Production for FUS, EWSR1, and Fusions With Different Truncations and Mutants

Protein	Solubility	Purity	Stability*^a^*	Yield
H‐MBP‐FUS	High	High	Stable	High
H‐MBP‐FUS‐LC	Medium	High	Stable	High
H‐MBP‐EWSR1	Medium	Medium	Stable	Medium
H‐MBP‐EWS‐del‐LC	High	Medium	Stable	Medium
H‐MBP‐EWS‐R_1_‐RRM‐R_2_	High	High	Stable	High
H‐MBP‐EWS‐R_2_‐ZnF‐R_3_	High	High	Stable	High
H‐MBP‐EWS‐FLI1	Poor	Poor	Unstable	Low
H‐MBP‐EWS‐ETV1 (trunc)	Poor	Poor	Unstable	Low
H‐MBP‐EWS‐ETV1_LCdead	Poor	Poor	Unstable	Low
H‐MBP‐EWS‐ETV1_DBDnull	Poor	Poor	Unstable	Low

^*a*^ Stability refers to apparent amount of full‐length protein relative to products that are truncated or degraded while in cells.

### Critical Parameters and Troubleshooting

The most important parameters for this protocol center on protein expression and lysis. In our experience, low protein expression, degradation, or insolubility are most likely to occur irreparably before or during cell lysis (step 21 in Basic and Alternate Protocols). After lysis, troubleshooting steps are usual minor adjustments addressed in product manufacturer instructions, such as for Ni‐NTA beads. For a list of common problems associated with these protocols and possible solutions to them, please refer to Table 4.

**Table 4:** Troubleshooting Common Problems Purifying FET or FET‐Fusion Proteins

Problem	Possible cause	Solution
Low expression or truncated protein	Protease activity in *E. coli*	Express protein overnight at 17°C
Low expression	Plasmid backbone	We use a pGEX‐6p‐1 backbone for expression; we have noted significant lower protein expressed from backbones including pET28 for this protocol
	Poor lysis	Optimize lysis to ensure protein is not lost in unlysed *E. coli* while clearing lysates
Protein precipitate in lysate	Solubility tag	MBP tag is ideal
	Nucleic acid binding	A minimum of 0.5 M urea lessons RNA binding and aggregation
	Precipitate seeded by heat or sonication	Sonication is convenient, but too much or heating will cause precipitate; a good alternative is a high‐pressure homogenizer, e.g., Emulsiflex
Loss of activity or protein concentration during storage	Soluble or insoluble aggregation	Most FET and FET fusion proteins store poorly frozen or at 4°C; store tagged protein at room temperature and 1 M urea
	Aging	FET proteins can maintain activity for ≥1 month in storage; we find it more convenient to replace any FET protein by 6 months after purification, if not sooner

#### Protein expression

The strains of *E. coli* used in this protocol can be BL21 or BL21(DE3), depending on requirements of the expression plasmid. These strains are ideal for FET protein preparation because they are optimized for protein expression and competent cells are obtainable from commercial sources or through laboratory preparation. In unpublished tests performed to date, we have not found improvement in protein yield or solubility from specialized *E. coli* strains, such as Rosetta(DE3) and Origami(DE3). As described above, plasmid design should be carefully considered, as the promoter, tags, selection markers, and specific modifications to the protein of interest are important for success. Our work has mostly relied on a modified pGEX‐6P‐1 backbone with a *tac* promoter, a hybrid of the *trp* and *lac* UV5 promoters. The *tac* promoter drives high FET protein expression and is compatible with both BL21 and BL21(DE3) strains of *E. coli*.

We often use a low temperature and overnight incubation to induce protein expression. Due to their disordered domains and tendency to cause toxicity in *E. coli*, recombinant nucleic acid binding proteins are frequently expressed at low temperatures. This is especially true for the ETS family proteins, whose DNA‐binding domains, when recombinantly expressed, are not particularly well‐folded and thus susceptible to proteases (Hou et al., 2021; K. M. Johnson et al., 2017). We have observed that expression of FUS or EWSR1 at 37°C or room temperature can halt *E. coli* growth by 3 hr and thereby does not provide the same protein yield as overnight incubation at 17°C. We have not found improvement in ETS or ETS‐fusion protein stability when expressed at 14°C, rather than 17°C. There is no difference in the activities of FUS or EWSR1 expressed at 37°C for 3 hr or 17°C overnight.

#### Optimized lysis buffer

Several factors are critical when selecting a lysis buffer, including pH, ionic strength, detergents, denaturants, and additives to enhance protein stability (Peach et al., 2015). While other laboratories have described lysis buffers for specific protein types (Gromov et al., 2008), a universally applicable lysis buffer suitable for a wide range of proteins remains elusive. Consequently, most lysis buffers and protocols are tailored to the specific requirements of individual proteins. The lysis buffers used in this study have been optimized with these considerations in mind.

Two key challenges specific to FET and FET‐fusion proteins are their propensity to aggregate and the role of nucleic acids in promoting aggregation. Both issues can be mitigated by including moderate concentrations of urea (1 to 2 M) in the lysis and storage buffers, as described above. Published literature and our findings indicate that these urea concentrations do not destabilize the ZnF or RRM domains of FET proteins. Urea is particularly helpful to ensure removal of trace amounts of co‐purifying nucleic acids.

Standard lysis buffer components do not appear to adversely affect protein yield or stability for FET proteins and FET fusion proteins. These components include salt concentrations up to 1 M, nonionic detergents at 0.1% to 2%, ionic detergents at 0.01% to 0.5%, divalent cation concentrations up to 10 mM, EDTA concentrations up to 5 mM, and pH ranges between 6 and 8. We have successfully purified FUS using lysis buffers containing 0.5 to 1 M KCl; lower yields were observed at 0.25 M KCl. While we have tested nonionic detergents, such as NP‐40 (0.5%) and Triton X‐100 (1%), we did not find clear evidence that they improve protein solubility or yield.

**Figure 3:**
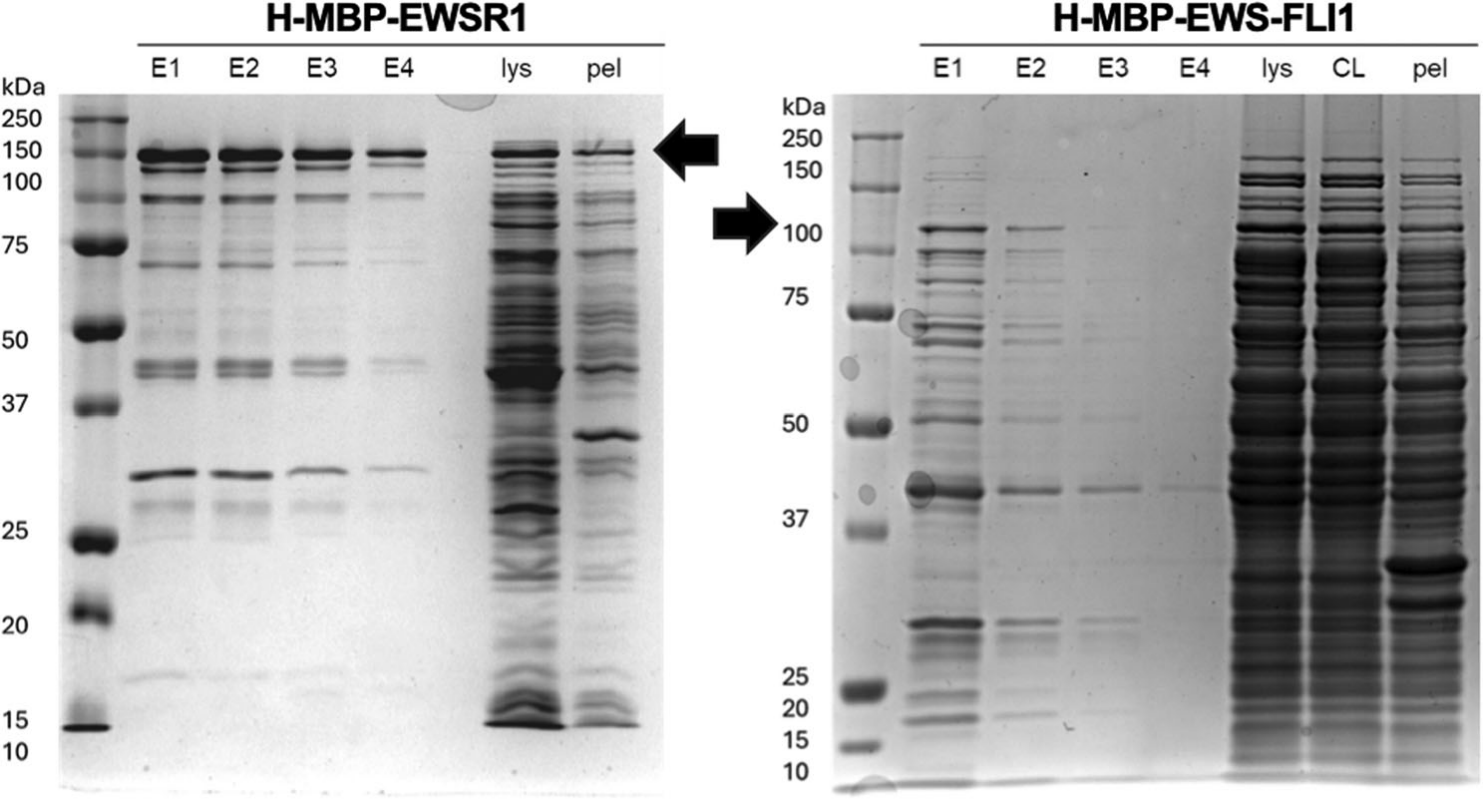
SDS‐PAGE of purified proteins. Ni‐NTA bead affinity purification. E1‐E4, elutions 1‐4; Lys: cell lysate prior to affinity purification; CL, cleared cell lysate after affinity purification; Pel, pellet after centrifugation of insoluble proteins. Arrows indicate expected molecular weight for H‐MBP‐EWSR1, left, and H‐EWS‐FLI1, right.

Although the inclusion of protease inhibitors in lysis buffers is a common practice, particularly for disordered proteins, our observations suggest that the truncation of FET and FET fusion proteins predominantly occurs prior to cell lysis. We found that full‐length FUS is consistently purified with minimal to no truncated products (Fig. 2). For most preps of EWSR1 some cleaved protein is observed (Fig. 3). EWS‐FLI1 undergoes considerable C‐terminal truncation that co‐purifies due to the N‐terminal 6×His tag (Fig. 3).

#### Monitoring protein stability and activity

The loss of FET and FET fusion proteins occurs primarily due to aggregation or oligomer‐forming conditions (K. M. Johnson et al., 2017). Key factors are low temperatures, high protein concentrations, and trace amounts of nucleic acids. We find that protein aggregation increases, and activity rapidly decreases with storage at −80°C. FET protein solubility and activity declines fastest when stored at 4°C or −20°C. We have found that storage at room temperature is best for FET and FET‐fusion proteins (Lay et al., 2024; Thompson et al., 2023).

Evidence of FET and FET‐fusion protein aggregation can be seen as a cloudy or white precipitate. Once clarified by centrifugation at maximum speed, the protein concentration in solution is usually not much reduced. However, this is not true if stored at 4°C or −20°C, which converts at least 70% of soluble FET protein into an irreversible precipitate. We have found long term storage of FET protein is best as pellets of induced and unlysed *E. coli* flash frozen in liquid nitrogen and stored at −80°C. We find that yield and activity of FUS protein is stable for pellets stored for 6 months to a year at −80°C. For EWSR1, we find activity is only maintained if the *E. coli* pellet has been stored at −80°C no more than 3 months.

To assess protein activity, we use a variety of assays. The most robust activity of FET proteins is phase separation of monomer protein into either condensates or hydrogels, which is most often assessed by turbidity or through microscopy (Burke et al., 2015; Forman‐Kay et al., 2022). The second most enduring activity is RNA and DNA binding. We have found that RNA binding activity for FUS and its truncations can stay consistent for 6 months or more when stored at room temperature. For EWSR1, nucleic acid binding activity declines considerably after 1 month of storage. The most sensitive activity we have studied is the effect on transcription. We find FUS and its truncations typically lose activity after 2 or 3 months of storage.

### Time Considerations

Growth of *E. coli* from transformation to lysis of cells takes ∼3 days. Lysis and purification of protein takes ∼3 to ∼4 hr. PAGE, Coomassie staining, and imaging takes ∼3 hr.

### Author Contributions

**Jesse J. Altemus**: Writing–original draft; writing–review and editing; investigation; formal analysis. **Michelle A. Lay**: Writing–original draft; writing–review and editing; investigation; formal analysis. **Valery F. Thompson**: Writing–review and editing; formal analysis; methodology. **Jacob C. Schwartz**: Writing–review and editing; formal analysis; funding acquisition; supervision.

### Conflict of Interest

The authors declare no conflict of interest.

## Data Availability

Data sharing not applicable as no datasets were generated or analyzed during the current study. The expression plasmids are available from the authors upon reasonable request.
